# Engineering Alendronate‐Composed Iron Nanochelator for Efficient Peritoneal Carcinomatosis Treatment

**DOI:** 10.1002/advs.202203031

**Published:** 2022-09-04

**Authors:** Jing Zhao, Xiuyu Huang, Peng Liu, Miaojuan Qiu, Binbin Li, Yingfei Wen, Yongshu Li, Qiang Wang, Meiying Wu, Yu Chen, Yihang Pan

**Affiliations:** ^1^ Precision Medicine Center Scientific Research Center The Seventh Affiliated Hospital Sun Yat‐sen University Shenzhen 518107 P. R. China; ^2^ School of Pharmaceutical Sciences (Shenzhen) Shenzhen Campus of Sun Yat‐sen University Shenzhen Guangdong 518107 P. R. China; ^3^ Materdicine Lab School of Life Sciences Shanghai University Shanghai 200444 P. R. China

**Keywords:** calcium overload, chemotherapy, infinite coordination polymers, iron chelator, ovarian cancer

## Abstract

Iron is an essential element for various cellular metabolism. Cancer cells also have high requirement of iron in their proliferation, invasion, and metastasis processes. Alendronate (ALN), a kind of FDA‐approved bisphosphonates with metal‐chelating capability, is initially certified to selectively bind to intracellular Fe^3+^ theoretically and experimentally in this study. Hence, CaALN iron nanochelator is rationally designed to kill cancer cells by synergism of Fe‐depletion and calcium accumulation. In vitro experiments and RNA sequencing analysis indicate that CaALN nanomedicine inhibits the proliferation of cancer cells by depleting Fe, interfering with DNA replication, and triggering intracellular reactive oxygen species (ROS). Meanwhile, released Ca^2+^ and ROS mutually promote and induce damage of cellular macromolecules, which leads to mitochondrial apoptosis of cancer cells. In an intraperitoneal disseminated mouse model with the human ovarian cancer cells SKOV3, CaALN nanoparticles selectively accumulate in tumor tissues and result in significant retardation of tumor growth and ascites formation. The mean survival time of SKOV3‐bearing mice in treatment group is prolonged from 33 to 90 d. These results indicate that the alendronate‐originated iron chelator can serve as an efficient strategy for the treatment of peritoneal carcinomatosis.

## Graphical abstract

An alendronate‐composed iron nanochelator is designed for peritoneal carcinomatosis treatment. Satisfactory antitumor effect is achieved by synergism of Fe‐depletion and calcium accumulation, which promote mutually and eventually leads to the apoptosis of cancer cells.

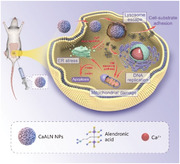

## 1 Introduction

Iron is an essential requirement for both human normal cells and cancer cells. It plays crucial roles in many biological processes, such as cellular metabolism, DNA synthesis and repair, oxygen transport, and energy production.^[^ ^1^ ^]^ It has been confirmed that several cancer cells have higher transferrin receptor 1 (TfR1) expression than normal cells because they have high requirements of iron to support their rapid growth, proliferation, and metastasis.^[^ ^2^ ^]^ Thus, targeting iron metabolism in cancer cells (Fe‐deficiency or ferroptosis) represents a new treatment strategy for cancers.^[^ ^3^ ^]^ Several small molecule iron chelators, such as deferoxamine (DFO), deferasirox (DFX), and some polyphenols were reported to preferentially reduce cancer cell growth and reduce tumor burden in vitro and in vivo.^[^ ^4^ ^]^ In addition, iron depletion, mostly using of DFO, was proved as a potential strategy for clinical cancer treatment.^[^ ^5^ ^]^

Ovarian cancer is one of the most lethal gynecological cancers and characterized by diffuse peritoneal metastasis.^[^ ^6^ ^]^ Intraperitoneal chemotherapy has been demonstrated to be very effective for the treatment of ovarian cancer with peritoneal metastasis, which can prominently increase the drug concentration in the abdominal cavity with reduced systematic toxicity.^[^ ^7^ ^]^ The current first‐line drugs for intraperitoneal perfusion are mainly antineoplastic drugs which are designed for intravenous injection, such as platinum‐based drugs, 5‐fluorouracil, paclitaxel, etc.^[^ ^8^ ^]^ However, due to the lack of tumor specificity and low bioavailability, the efficacy of small molecule based intraperitoneal chemotherapy is severely compromised.^[^ ^9^ ^]^ It has been reported that not only the level of Fe in serum and tumor tissues in advanced‐stage increased as compared with early‐staged ovarian cancer patients, but also proteins related to Fe uptake, such as transferrin receptor 1 (TFR1), ferritin, divalent metal ion transporter 1 (DMT1) increased in ovarian cancer tissues.^[^ ^10^ ^]^ Thus, it is plausible to believe that targeting Fe metabolism may be an effective therapeutic strategy for ovarian cancer.

Bisphosphonates, with the P‐C‐P bond in their structure, have been extensively used in the treatment of osteoporosis and skeletal metastases due to their low toxicity, high thermostability and inhibition of bone resorption.^[^ ^11^ ^]^ In the last two decades, nitrogen‐containing bisphosphonates have been used in clinical for the adjuvant treatment of breast cancer and prostatic cancer to reduce the incidence of skeletal‐related events.^[^ ^12^ ^]^ Alendronate, one of the nitrogen‐containing bisphosphonates, has been reported to exert an antiangiogenic effect and inhibit the invasiveness of human ovarian cancer cells. Current reports of alendronate were focused mainly on the antiangiogenic effect by inhibiting enzymes of the mevalonate pathway.^[^ ^13^ ^]^ In fact, bisphosphonates have also been proposed as potent chelators for metallic ions. The coordination of alendronate with metallic ions, especially Ca (II), has been exclusively studied.^[^ ^14^ ^]^ ALN‐functionalized polymers and nanoparticles have been reported as carriers for bone‐targeting drug delivery.^[^ ^15^ ^]^ But few works studied alendronate on its homeostasis of intracellular essential metallic ions yet. On the other hand, alendronate small molecule is not a desirable anticancer medicine due to its unfavorable pharmacokinetics. After intravenous administration of alendronate salts, the majority of alendronate binds to the mineralized bone matrix, and the remainder is excreted by the kidney.^[^ ^11^, ^16^ ^]^

In recent years, nanoformulations have been demonstrated as a viable strategy to solve the problems associated with conventional chemotherapeutic agents by the composition of nanotechnology, biomedicine, and pharmaceutical science, thus achieving reduced system toxicity, increased tissue selectivity, and enhanced therapeutic efficacy.^[^ ^17^ ^]^ Nevertheless, clinical applications of nanomedicine are limited by the low drug‐loading efficiency and immunogenicity of carriers.^[^ ^18^ ^]^ Infinite coordination polymers (ICPs) are amorphous compounds bearing polydentate ligands and metal ions, exhibiting higher tailorability and degradation than the regular crystalline coordination polymers.^[^ ^19^ ^]^ Therapeutic agents, such as methotrexate, oxaliplatin, gallic acid, and doxorubicin have been reported to coordinate with metal ions forming ICPs, showing superior antitumor efficacy and slight side effects.^[^ ^20^ ^]^

In this work, we reported for the first time that alendronate inhibited cancer cell proliferation by interference with intracellular iron metabolism. Alendronate‐based nanomedicine (CaALN) was rationally designed as iron nanochelator to deplete intracellular iron. In vitro study revealed that CaALN nanoparticles not only chelated intracellular iron and perturbed DNA replication, but also disturbed Ca^2+^ homeostasis, which induced intracellular ROS elevation and mitochondrial apoptosis of cancer cells. RNA sequencing has indicated that Fe deficiency and apoptosis related pathways were upregulated, while DNA replication and cell‐migration related pathways were downregulated. In an intraperitoneal xenograft model with SKOV3 cells, CaALN prominently suppressed the tumor progression and ascites formation. Generally, alendronate‐based iron nanochelator features considerable potential in clinical peritoneal carcinomatosis treatment.

## 2 Results and Discussion

### 2.1 Specific Binding of Alendronate with Fe and Synthesis of ICPs

**Figure 1:**
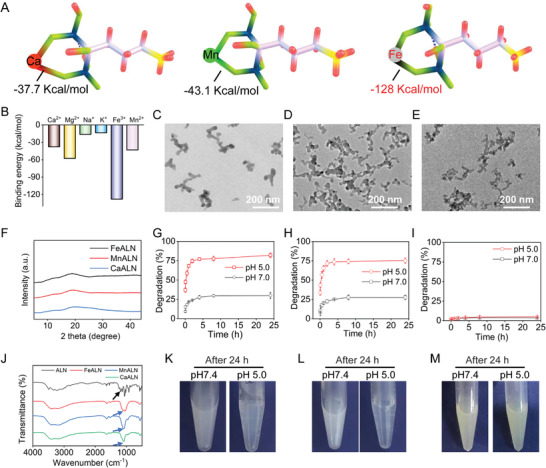
Characterization of alendronate‐assembled ICPs. A) Schematic illustration of coordination between alendronate and Ca^2+^, Mn^2+^, and Fe^3+^ in normal saline (pH 7.0). B) Calculated binding energy of alendronate and metal ions in normal saline (pH 7.0). TEM images of C) CaALN, D) MnALN, and E) FeALN NPs. F) XRD patterns of CaALN, MnALN, and FeALN. G) Degradation of CaALN in PBS with different pH values (7.0 or 5.0). H) Degradation of MnALN in PBS with different pH values (7.0 or 5.0). I) Degradation of FeALN in PBS with different pH values (7.0 or 5.0). J) FTIR spectra of NaALN, CaALN, MnALN, and FeALN. The photographs of K) CaALN, L) MnALN, and M) FeALN after 24 h incubation in PBS with different pH values (7.0 or 5.0).

Alendronate, a clinically approved drug for the treatment of osteolytic metastases,^[^ ^21^ ^]^ has been reported to have high efficiency in binding with metal ions. However, the interference of alendronate on intracellular ions metabolism was rarely investigated. In this study, chemical binding energies of alendronate and several intracellular metallic ions were obtained by density functional theory (DFT) calculation. As show in **Figure** **1**A and Figure S1 (Supporting Information), alendronate could chelate intracellular metal ions through the coordination between O atoms of phosphate and metals. Among the six types of common intracellular metal ions, Fe displayed the strongest binding with alendronate (Figure 1B), reminding us that alendronate may be a potential Fe chelator to disrupt Fe metabolism of cancer cells. To verify the combination of alendronate and metal ions, alendronate‐based ICPs were achieved by the quick reaction of alendronate and metal ions at 4 °C for 30 min. The obtained CaALN, MnALN, and FeALN ICPs exhibited rodlike morphology with amorphous states (Figure 1C–F). Energy dispersive X‐ray (EDX) spectrometer and elemental mapping recognized the existence of metal elements and phosphonate in ICPs (Figures S2–S5, Supporting Information). The nitrogen–adsorption–desorption isotherms and low specific surface areas also confirmed their disordered interior structures (Figure S6, Supporting Information). The broad absorption band centered at 1100 cm^−1^ (metal—O—P stretching vibration, blue arrow) and disappeared peak centered at 1235 cm^−1^ (P=O stretching vibration, black arrow) in Fourier transform infrared (FTIR) spectra confirmed the covalent binding of metal ions to phosphate (Figure 1J).^[^ ^21^, ^22^ ^]^ The positive zeta potentials (19.1 ± 0.83 mV for CaALN, 12.06 ± 0.10 mV for MnALN, and 24 ± 1.38 mV for FeALN) could be attributed to the amino group in alendronate, which further verified the successful coordination of alendronate and metal ions. Figure 1G–I showed the pH‐triggered degradation behaviors of CaALN and MnALN, while the degradation rate of FeALN was less than 5% at both pH 7.0 and 5.0 (Figure 1K–M). These results together demonstrated that alendronate had much higher affinity to Fe rather than other metallic ions. However, the high affinity to bone tissue limits its clinical application as a versatile antitumor drug.

### 2.2 Intracellular Fe‐Depletion of NaALN and CaALN in Cancer Cells

**Figure 2:**
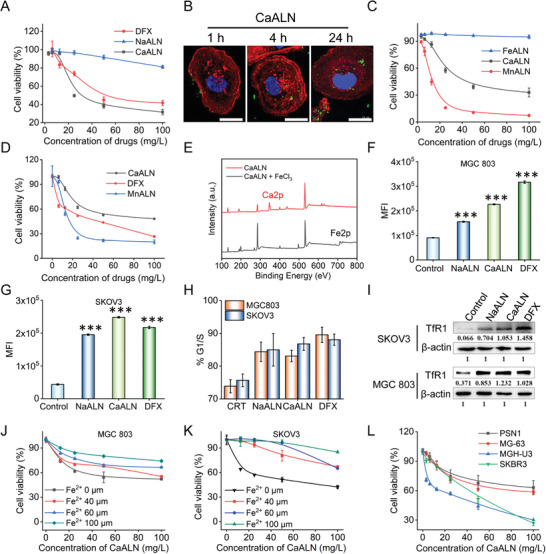
In vitro Fe‐depletion abilities of NaALN and CaALN in SKOV3 and MGC803 cells. A) Relative viabilities of SKOV3 cells after treatment with various concentrations of NaALN, CaALN, and DFX for 48 h. B) Abberior STEDYCON super‐resolution images of SKOV3 cells incubated with CaALN NPs at different time points, bar = 20 µm. C) Relative viabilities of SKOV3 cells after treatment with various concentrations of FeALN, CaALN, and MnALN for 48 h. D) Relative viabilities of human renal tubular epithelial cell HK2 after treatment with various concentrations of DFX, CaALN, and MnALN for 48 h. E) XPS analysis of CaALN powder before and after immersing in FeCl_3_ solution for 24 h. Detection of intracellular Fe concentration using calcein‐AM in F) MGC 803 and G) SKOV3 cells after treatment with NaALN, CaALN, and DFX at 25 mg L^−1^ for 24 h by FCM analysis (mean ± SD, *n* = 3, ***P < 0.001 vs control group). H) Cell cycle distributions of G1/S phase in MGC803 and SKOV3 cells treated with NaALN (25 mg L^−1^), CaALN (25 mg L^−1^), and DFX (25 mg L^−1^) for 24 h and analyzed by FCM. I) Western blot analysis of MGC 803 and SKOV3 cells treatment with NaALN, CaALN, and DFX at concentration of 25 mg L^−1^ for 24 h. Treatment of J) MGC803 and K) SKOV3 cells with CaALN in the presence or absence of FeSO_4_ for 48 h. L) Relative viabilities of PSN1, MG‐63, MGH‐U3, and SKBR3 cells after treatment with various concentrations of CaALN for 48 h.

Herein, we report a nanoformulation strategy of alendronate to improve its tumor targeting and anticancer potency. **Figure** **2**A showed that at equipotent concentration, CaALN nanoparticles revealed the strongest cytotoxicity on SKOV3 cells, followed by the commercial Fe‐chelator DFX. This result might be attributed to the synergistic effect of alendronate and Ca^2+^. Moreover, it is noteworthy that CaALN was ≈150 nm in size and positive charged (19.1 ± 0.83 mV), which makes CaALN well adapted for cancer cell uptake (Figure 2B and Figure S7, Supporting Information). Likewise, MnALN also exhibited potent anticancer ability, while FeALN featured no cytotoxicity due to its undegradability in physiological environments (Figure 2C). Figure 2D showed that CaALN revealed relatively lower cytotoxicity than MnALN and DFX in normal human epithelial cell (HK2). The serum calcium concentration of normal human is 2.25–2.75 mmol L^−1^, which is higher than the serum manganese concentration (0.073–0.255 mmol L^−1^), demonstrating Ca ‐baesd material is more tolerable for human body. Considering the excellent biocompatibility and clinical availability of Ca‐based biomaterials, CaLAN was eventually optimized for human ovarian cancer treatment. Next, the chelation exchange behavior of CaALN in Fe^3+^ solution was studied. As shown in Figure S8 (Supporting Information), white CaALN sample turned into yellow after immersing in FeCl_3_ solution for 24 h. X‐ray photoelectron spectroscopy (XPS) test was performed to study the chemical composition of these powders. As shown in Figure 2E, disappearance of Ca2p peak at 359 eV and emergence of Fe2p peak at 739 eV revealed that most Ca has been replaced by Fe. The intracellular Fe‐chelation ability of NaALN and CaALN was further investigated by an established calcein method.^[^ ^23^ ^]^ In brief, the free iron in cytosol and labile iron pool (LIP) can combine with carboxyl group of calcein AM and quench the fluorescence of calcein AM. After co‐incubation with powerful Fe chelating agents, the fluorescence intensity of calcein AM could be recovered. As shown in Figure 2F,G, in comparison with DFX, treatment with NaALN and CaALN revealed analogous Fe‐depletion ability in MGC803 and SKOV3 cells. As Fe is critical for DNA synthesis, Fe deficiency would inhibit the activity of ribonucleotide reductase,^[^ ^24^ ^]^ the cell cycle distributions of cancer cells were detected by flow cytometry (FCM). Figure 2H showed obvious G1/S cell cycle arrest after NaALN and CaALN treatment in MGC803 and SKOV3 cells, which was similar with DFX treatment. Moreover, NaALN and CaALN treatment increased the expression of TfR1 protein and Fe^2+^ supplement could promote the recovery of cancer cells after treatment of CaALN, implying that Fe‐deficiency was one of the action modes of CaALN in cancer cell (Figure 2I–K). In addition, CaALN exhibited broad‐spectrum antitumor activity, the cytotoxicity of CaALN against human pancreatic tumor cell PSN1, human osteosarcoma cell line MG63, human breast cancer cell SKBR3 and human bladder cancer cell MGH‐U3 were shown in Figure 2L.

### 2.3 Synergetic Anticancer Effect of Alendronate and Ca^2+^

**Figure 3:**
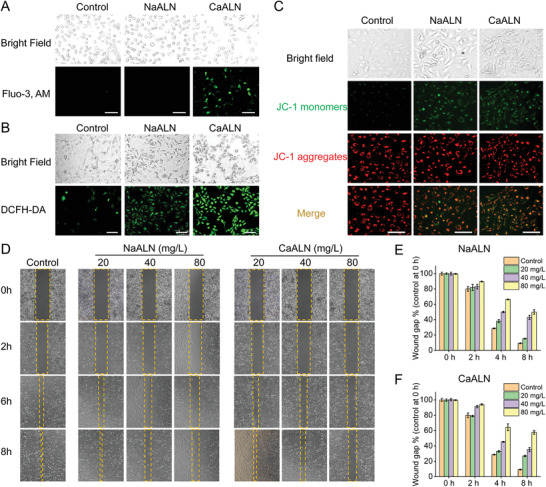
In vitro anticancer evaluation of alendronate‐assembled ICPs. Fluorescence microscope images of SKOV3 cells treated with CaALN for 24 h and stained with A) Fluo‐3, AM, B)DCFH‐DA, and C) JC‐1 , scale bar = 100 µm. D) Microscope images and E,F) corresponding quantitative analyses of wound healing in SKOV3 cells treated with NaALN and CaALN at different concentrations. The wound gap was calculated as dehiscence/initial dehiscence × 100%.

The underlying anticancer mechanism of CaALN on SKOV3 cells was further studied. Ca^2+^, as an important second messenger, plays essential roles in a range of cellular processes such as gene expression, cell proliferation, and apoptosis. Intracellular Ca^2+^ level is precisely regulated by an effective Ca^2+^ homeostatic system, which is 10 000 times lower than that of the extracellular fluid, thus both excess and deficiency of Ca^2+^ in cells may result in undesirable cytotoxicity. Co‐incubation of CaALN could cause calcium accumulation in SKOV3 cells, as detected by intracellular calcium fluorescent probe (**Figure** **3**A and Figure S9, Supporting Information). Thus, the Ca^2+^ retention synergized with alendronate to induce high ROS generation in cancer cells (Figure 3B and Figure S10, Supporting Information), which leads to the oxidative damage to membrane systems, including mitochondria. On the other hand, it has been reported that iron deficiency decreased mitochondrial function.^[^ ^25^ ^]^ Herein, the mitochondrial membrane potential (MMP) of SKOV3 cells after being treated with NaALN or CaALN was assessed using JC‐1 fluorescence probe (Figure 3C). The remarkably enhanced green/red fluorescence intensity ratio stated the significant mitochondrial membrane potential loss and mitochondrial injury caused by NaALN or CaALN. Finally, Ca^2+^ accumulation, ROS generation, and mitochondrial dysfunction jointly resulted in the apoptosis of SKOV3 cells (Figure S11, Supporting Information). In addition, alendronate indicated to prevent cancer cell migration in vitro and in vivo by inhibiting the mevalonate pathway.^[^ ^13^, ^26^ ^]^ The wound healing assay showed that both NaALN and CaALN could effectively inhibit in vitro migration of SKOV3 cells in a time‐ and dose‐dependent manner (Figure 3D–F), suggesting that CaALN nanoparticles would inhibit cancer growth via multiple mechanisms.

### 2.4 RNA Sequencing Analysis of CaALN against SKOV3 Cells

**Figure 4:**
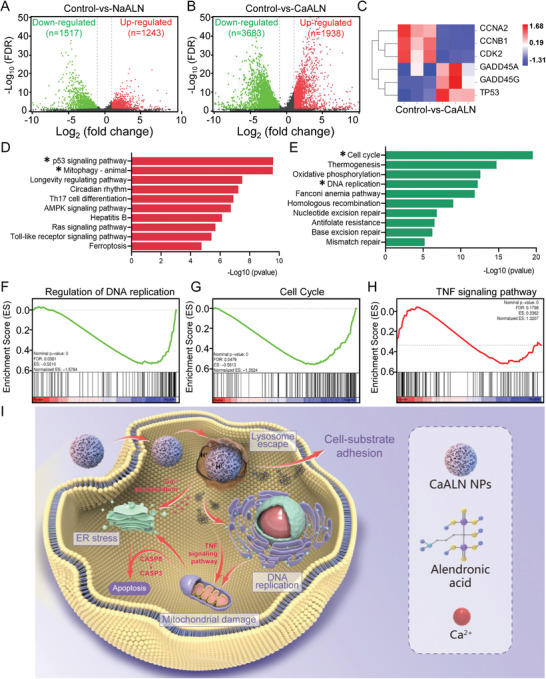
RNA sequencing analysis of SKOV3 cells treated with NaALN or CaALN. Volcano plots for all the expressed genes of control versus A) NaALN and B) CaALN treatments. Red (upregulated) and green (downregulated) dots mean that the genes have significant differences (FDR ≤ 0.001, and at least with twofold differences). C) Heat map of differentially expressed genes related to intracellular Fe‐deficiency. D,E) The top 10 remarkably enriched pathway of upregulated and downregulated of differentially expressed genes by KEGG analysis. GSEA enrichment plots of differentially expressed genes centralized in F) regulation of DNA replication, G) cell cycle, and H) TNF signaling pathway. I) Schematic illustration of anticancer mechanism of CaALN in SKOV3 cells.

We further conducted RNA sequencing analysis to assess the regulation of gene expression and analyze differentially expressed genes. Statistically significant genes with a log_2_ (fold change) expression of ≥1.0 were considered to be upregulated and ≤−1.0 were considered to be downregulated (treated group vs control group). RNA sequencing analysis showed that compared with the cells in the control group, there were significant changes in mRNA abundance of 1243 upregulated and 1517 downregulated genes in cells treated with NaALN (**Figure** **4**A), while 1938 upregulated and 3683 downregulated genes in cells treated with CaALN (Figure 4B). Moreover, CaALN and NaALN treated cells shared 973 upregulated and 1338 downregulated genes (Figure S12, Supporting Information). Fe‐deficiency was reported its significant effects on cell cycle and cell metabolism.^[^ ^27^ ^]^ As shown in Figure 4C, cell cycle‐associated genes, including cyclin A2 (CCNA2), cyclin B1 (CCNB1), and cyclin‐dependent kinases 2 (CDK2) were downregulated after treatment with CaALN. Meanwhile, TP53 tumor suppressor gene and its downstream genes, growth arrest‐ and DNA damage‐inducible genes (GADD45A and GADD45G) were upregulated. We subsequently applied a bioinformatics approach to explore the upstream regulators and functional significance of such alterations according to the identified differential expression genes. Kyoto Encyclopedia of Genes and Genomes (KEGG) enrichment analysis revealed the top 10 upregulated (Figure 4D) and downregulated pathway (Figure 4E). Therein, p53 signaling pathway and Ras signaling pathway, which were involved in cell proliferation and apoptosis, were prominently upregulated. The upregulation expression of P53 protein and key genes on TNF signaling pathway were shown in Figures S13 and S14 (Supporting Information). The activation of RAS pathway might be attributed to the acquired and intrinsic resistance of cancer cells in chemotherapy.^[^ ^28^ ^]^ Meanwhile, the upregulated mitophagy confirmed the mitochondria damage, consistent with the result of the JC‐1 fluorescence probe in Figure 2A. In addition, Gene Set Enrichment Analysis (GSEA) of the downregulated genes in the DNA replication and cell cycle was further confirmed, which indicated that the treatment of CaALN induced SKOV3 cell apoptosis by removing iron, interfering with the cell cycle and activating TNF‐mediated caspase‐dependent pathway (Figure 4F–H). Upregulation of key genes enriched in the TNF signaling pathway were further confirmed by qRT‐PCR analysis, including upregulation of the *Tumor Necrosis Factor Receptor Type 1 Associated Death Domain Protein (TRADD)*, *Apoptosis‐Related Cysteine Peptidase (Caspase8), Receptor Interacting Serine/Threonine Kinase 1(RIPK1)*, and *Receptor Interacting Serine/Threonine Kinase 3 (RIPK3)* gene (Figure S15, Supporting Information). Based on those results, a possible antitumor mechanism of CaALN against SKOV3 cells was shown in Figure 4I. After being internalized by tumor cells, CaALN could induce cell cycle arrest, ROS elevation, and mitochondrial damage, which eventually leads to caspase‐dependent apoptosis of SKOV3 cells.

### 2.5 In Vivo Biodistribution of CaALN Nanomedicine

**Figure 5:**
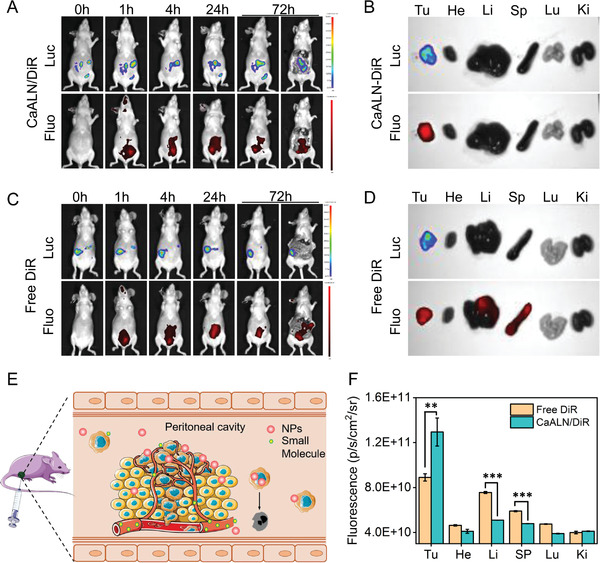
In vivo biodistribution of CaALN. In vivo time‐dependent bioluminescence and fluorescence images of SKOV3 tumor‐bearing mice after i.p. injection of A) CaALN/DiR and C) free DiR i.p. treatment. B,D) In vitro bioluminescence and fluorescence images of tumors (Tu), livers (Li), hearts (He), spleens (Sp), lungs (Lu), and kidneys (Ki) dissected from SKOV3 tumor‐bearing mice post 72 h i.p. injection. E) Illustration of in vivo distribution of CaALN nanoparticles and small molecules. F) Fluorescence intensity of in vitro tissues from mice treated with CaALN/DiR and DiR (mean ± SD, *n* = 3, ***P < 0.001, **P <0 .01).

Although alendronate exhibited strong antineoplastic ability in vitro, the bone affinity hindered its clinical applications as an antineoplastic drug. In this study, alendronate coordinated with Ca^2+^ to improve its tumor targeting efficiency. To evaluate the in vivo distribution of CaLAN, an intraperitoneal disseminated tumor xenograft model was established by intraperitoneal injection of 5 × 10^6^ SKOV3 cells into NOD/SCID mice. It is noted that SKOV3 cells were stably transfected with lentivirus carrying luciferase (Luc) gene, which could exhibit strong bioluminescent signal and be captured by the In Vivo Imaging System(IVIS, AniView 100). To enable in vivo real‐time monitoring, CaALN nanoparticles were labeled by DiR fluorescence. As shown in the first row of **Figure** **5**A, luciferase signals in the abdominal cavity could be detected, implying successful establishment of intraperitoneal xenograft mice model. After 14 d of tumor establishment, the in vivo distribution of CaALN nanoparticles was evaluated by i.p. injection of CaALN/DiR or free DiR into mice. The images and quantitative fluorescence intensities of tumors and organs confirmed that i.p. injection of CaALN nanoparticles could target tumor tissue post 4 h injection (Figure 5A,B). In the free DiR group, fluorescence distributed not only in the tumor sites, but also in the liver and spleen (Figure 5C,D). The tumor targeting advantage of CaALN could be attributed to the existence of the peritoneal‐plasma barrier, which maintained a high regional concentration of CaALN nanoparticles in the peritoneal cavity and was eventually uptaken by cancer cells (Figure 5E). In comparison, small molecules could enter subperitoneal capillaries via the peritoneal–plasma barrier and metabolize by liver and spleen (Figure 5F).

### 2.6 In Vivo Anticancer Evaluation of CaALN Nanomedicine

**Figure 6:**
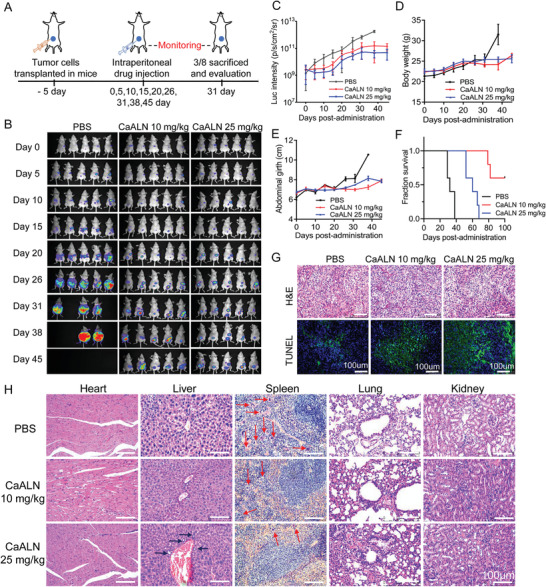
In vivo antitumor effect of CaALN on SKOV3 bearing mice. A) Scheme illustration of the in vivo antitumor experiment using SKOV3 intraperitoneal xenograft model. B) Bioluminescence images, C) bioluminescence intensity, D) body weight, E) abdominal girth, and F) survival curves of SKOV3 tumor‐bearing mice in each treatment group. G) H&E staining images and tunnel histochemical images of representative tumor sections at day 31. H) H&E staining images of organs from experimental mice.

Encouraged by the in vitro antitumor activity and in vivo tumor targeting capability, the in vivo tumor inhibition activity of CaLAN nanoparticle was further evaluated. Five days after tumor cell implantation, SKOV3‐bearing mice were treated with PBS and two different doses of CaALN (10 and 25 mg kg^−1^, *n* = 8). Five of the mice were periodically imaged and monitored for collecting data of body weight, abdominal girth, and survival day. The other three mice of each group were sacrificed at day 31 for real efficacy and biocompatibility evaluation (**Figure** **6**A). As shown in Figure 6B,C, both doses of CaALN‐treated groups maintained the bioluminescence by day 15, implying the complete suppression of tumor progression. The tumor inhibition effect of the high dose (25 mg kg^−1^) group was better than that of the low dose (10 mg kg^−1^) group. In comparison, treated mice began to exhibit abdominal swelling (Figure 6E) with ascites at 25 d post SKOV3 cell inoculation, the average survival day was 34 d (Figure 6F). At day 31, three mice of each group were sacrificed, the tumor burden and ascites were quantified, and the main organs were extracted for hematoxylin and eosin (H&E) dyeing. As shown in Figure S16 (Supporting Information), the mean tumor weight was 650.1 ± 135.4 mg in the PBS group, which was significantly reduced in CaALN treatment groups (238.8 ± 22.8 mg in the low CaALN dose group and 174.6 ± 24.1 mg in the high CaALN dose group). The ascites formation of mice was also inhibited by treatment of CaALN, where the mean weight of ascites in the PBS group was 3163 ± 450 mg, only 502 ± 154 mg in the low CaALN dose group, and 57.2 ± 8.5 mg in the high CaALN dose group (Figure S17, Supporting Information). Meanwhile, H&E and TUNEL staining also identified the severe cancer cell apoptosis and less tumor cell invasion (black arrow) in the spleen in both CaALN treatment groups (Figure 6G,H). Finally, the mean survival time in the CaALN treatment groups was significantly longer than the control group (Figure 6F).

After six cycles of treatment, the mice in all the CaALN‐treated groups showed stable body weights (Figure 6D). The body weight of the PBS group rose dramatically at day 38 due to the formation of massive ascites. However, H&E staining of organs revealed that in the high dose of CaALN group, the liver of mice had bile canaliculus proliferation (black arrows), suggesting of hepatic fibrosis. By contrast, the low dose CaALN group showed normal cellular morphology of organs. Therefore, from bioluminescence intensity, tumor and ascites weight results, high dose of CaALN induces the higher antiproliferation/antimigration activity of ovarian cancer in vivo. However, tumor‐bearing mice treatment with a low dose of CaALN exhibits longer survival due to its better biocompatibility. These results suggested that CaALN nanoparticles have considerable potential in treating peritoneal disseminated ovarian cancer at moderate concentration.

## 3 Conclusions

In summary, we have reported the selective Fe‐chelation ability of alendronate, based on which, an alendronate‐composed CaALN has been rationally designed for efficient peritoneal carcinomatosis treatment. In vitro experiments demonstrated that CaALN with pH sensitive degradation induced strong antiproliferation/antimigration activities in human cancer cell lines by causing Fe‐deficiency and cell cycle arrest. Importantly, exogenous Ca^2+^ aggravated mitochondrial dysfunction and intracellular ROS elevation, which involved in the apoptosis of SKOV3 cancer cells. Based on RNA sequencing analysis, we further confirmed that apoptosis related pathways were upregulated, and DNA replication, cell cycle and migration related pathways were downregulated. In an intraperitoneal xenograft model with SKOV3, it has been demonstrated that CaALN nanoparticles after i.p. administration accumulated mainly in tumor tissues. In addition, both doses of CaALN nanoparticles treatments (10 and 25 mg kg^−1^) not only decreased tumor burden, but also inhibited the formation of ascites, which presents a considerable clinical application for the management of ovarian cancer. The mice treated with a low dose of CaALN (10 mg kg^−1^) have longer lifetime with no obvious toxic effects observed. Generally, by using alendronate formed iron nanochelator, this study has demonstrated a distinct strategy to enhance the therapeutic potency and reduce the side effects of small molecule for efficient peritoneal carcinomatosis treatment.

## 4 Experimental Section

### Materials

CaCl_2_, MnCl_4_•4H_2_O, FeCl_3_•6H_2_O, alendronate sodium trihydrate, deferasirox (DFX), ethanol, and NaOH were purchased from Sigma‐Aldrich. 6‐FAM SE (Ex/Em = 495/519 nm) was bought from Fanbo Biochemicals Co., Ltd. DiR Iodide (DiIC18(7), Ex/Em = 748/780 nm) was bought from Yeasen Biological Technology Co., Ltd. All chemical reagents were analytically pure and used without further purification. Cell Counting Kit‐8 (CCK8 kit) and calcein acetoxymethyl ester (Calcein‐AM) were bought from MCE (MedChemExpress, Shanghai). Fluo‐3 AM ester was bought from US Everbright Inc. AO/EB assay kit and Mitochondrial Membrane Potential Detection Kit (JC‐1) were bought from LEAGENE. Antibodies against TFR1 and p53 were purchased from Abclonal. Human ovarian cancer cell line SKOV3 and Human Gastric Carcinoma Cell Line MGC803, human pancreatic tumor cell PSN1, human osteosarcoma cell line MG63, human breast cancer cell SKBR3, and human bladder cancer cell MGH‐U3 were purchased from the American Type Culture Collection (ATCC). Female nude mice (5–6 weeks) were bought from Guangdong Province Animal Center, China.

### Synthesis of ICPs

For preparing CaALN, 243 mg of alendronate sodium trihydrate was dissolved in 40 mL of deionized water. CaCl_2_ solution (0.1 m, 10 mL) was added to the above alendronate sodium solution. Then, the pH value of the mixture was adjusted to 7.0 by 0.05 m NaOH and stirred for 30 min at 4 °C in the water bath. CaALN nanoparticles were obtained by centrifugation and washing by ethanol. FeALN and MnALN were synthesized in a similar way. For in vitro imaging, 1 mg of CaALN and 0.1 mg of 6‐FAM SE reacted in ethanol in dark for 12 h. After dialysis, 6‐FAM‐CaALN nanoparticles were obtained. For in vivo real‐time imaging, CaALN/DiR was prepared by mixing CaALN and DiR for 12 h and dialysis for 24 h.

### Characterizations

The morphologies of as‐synthesized ICPs were examined with transmission electron microscope (TEM, JEM‐2100F) and element analysis was carried out with an energy‐dispersive X‐ray spectroscopy (XPS, Thermo Scientific K‐Alpha). X‐ray diffraction (XRD) was obtained by Rigaku Ultima IV X‐ray diffractometer. Inductively coupled plasma‐optical emission spectrometry (ICP‐OES, Agilent 700 Series, Agilent Technologies, USA) was used for the quantitative elemental analysis. FTIR spectra were conducted on a Nicolet 7000‐C spectrometer in the range of 400–4000 cm^−1^. Brunauer–Emmett–Teller (BET) specific surface areas were measured with a surface area and porosity analyzer (Micromeritics ASAP 2460). The particle size and zeta potential were detected by Zetasizer Nano Series (Malvern, USA)

### Calculations

The binding energy of metal ions and alendronate was carried out with the Gaussian 16 software. The B3LYP functional and 6‐311G(d) basis set were adopted for all calculations.^[^ ^29^ ^]^ The IEFPCM implicit solvation model was applied to account for the solvation effect of water.^[^ ^30^ ^]^ The DFT‐D3 dispersion correction with BJ‐damping was applied to correct the weak interaction to improve the calculation accuracy.^[^ ^31^ ^]^ Then, the interaction energies between metal ion and alendronate were calculated by the following formula: Δ*G* = *G*_complex_ − (*G*_metal_ + *G*_alendronate_).

### In Vitro Degradation

10 mL of ICP was dispersed in 10 mL of Ca^2+^ and Mg^2+^ free PBS. After different incubation time at 37 °C, 100 *µ*L of supernatant was collected by centrifugation and analyzed by ICP. The degradation rate was calculated according to the metal concentrations in the supernatant.

### Chelation Exchange of CaALN in Fe^3+^ Solutions

20 mg of CaALN were dispersed in 20 mL of deionized water. Then, 20 mL of FeCl_3_•6H_2_O solution (1 × 10^−3^ m) was added into CaALN and stirred for 24 h at 37 °C. After centrifugation and drying, the obtained yellow product was collected for XPS analysis.

### In Vitro Cytotoxicity Assays

SKOV3 and MGC803 cells were seeded in 96‐well plates and cultured at 37 °C under 5% CO_2_ for 24 h. Cells were incubated with treatment agents at series of concentrations. Fe^2+^ supplemental experiment was conducted after 2 h treatment of CaALN. After 48 h of incubation, the culture media was washed with PBS and replaced with CCK8 solution, and the OD values were recorded at the wavelength of 450 nm. AO/EB staining, mitochondrial membrane potential, intracellular calcium ions, and ROS generation were carried out following the instructions. The mean fluorescence intensities of fluorescence microscope images were calculated by ImageJ software.

### Measurement of the Cellular Labile Iron Pool (LIP)

The measurement of LIPs was performed as a described calcein‐AM method.^[^ ^32^ ^]^ Briefly, SKOV3 and MGC803 were treated with DFX, NaALN, or CaALN at 50 mg L^−1^ for 48 h. Then these cells were washed with PBS and incubated with calcein‐AM (0.5 × 10^−6^ m) for 15 min at 37 °C in the dark. Finally, cells were collected and analyzed by flow cytometry.

### Cell Cycle Analysis

SKOV3 and MGC803 cells were seeded in six‐well plates and treated with NaALN, CaALN, and DFX at concentration of 25 mg L^−1^ for 24 h. After rinsing with PBS, cells were harvested and fixed in 75% ethanol for 24 h at 4 °C. The fixed cells were centrifuged and washed twice with PBS, then incubated with propidium iodide (PI) solution containing RNase A at 37 °C for 30 min in the dark. After washing and centrifugation, cells were analyzed by flow cytometry.

### Wound Healing Assay

SKOV3 cells were seeded in six‐well plates at a density of 6 × 10^5^ cells/well. When the cells grew to 90% confluence, one wound per well was made using a tip and the floating cells were removed by PBS washing. Then, serum‐free media with different concentrations of NaALN or CaALN were added. After 2, 4, and 8 h of incubation, photographs were taken by microscope and the migration distance was measured.

### Cellular Uptake of CaALN by SKOV3 Cells

SKOV3 cells were seeded into confocal dishes and incubated with 6‐FAM‐CaALN at concentration of 20 mg L^−1^ for 1, 2, 4, and 24 h. Then, the cells were washed twice with PBS and stained by DAPI and DiR. The cells were observed by an Abberior STED Superresolution System (STEDYCON, Abberior Instruments, Germany).

### Western Blotting (WB)

Cells were incubated with NaALN, CaALN, and DFX for 48 h at concentration of 50 mg L^−1^ and proteins were collected after lysis and quantified by BCA assay. The retrieved proteins were separated by using SDS‐PAGE and analyzed by immunoblotting. Protein bands were quantified by ImageJ software.

### Quantitative Real‐Time PCR (qPCR)

The cells of each well were harvested separately after coincubation with CaALN for 24 and 48 h. Total RNA of cells was extracted by the RNAex Pro Reagent Kit (AG21102, Accurate Biotechnology, China). First‐strand cDNA was synthesized using Evo M‐MLV RT Premix for the qPCR kit (AG11706, Accurate Biotechnology, China). *qRT‐PCR* was performed using SYBR Green Premix Pro Taq HS qPCR Kit (AG11701, Accurate Biotechnology, China) on CFX96 Real‐Time System (BIO‐RAD, Singapore). mRNA expression levels were analyzed by the 2^−ΔΔCt^ method and compared to the control. The specific primers were purchased from Sangon Biotech Co., Ltd. (Shanghai, China) and the primers sequences are listed in Figure S18 (Supporting Information).

### RNA Extraction and Sequencing

SKOV3 cells were treated with NaALN (0.1 mg mL^−1^) or CaALN (0.1 mg mL^−1^) for 24 h. Three biological replicates were carried out for each group and total RNA was extracted by Trizol reagent kit (Invitrogen, USA) following the manufacturer's protocol.^[^ ^33^ ^]^ Agilent 2100 Bioanalyzer (Agilent Technologies, USA) was used to assess total RNA quality.^[^ ^34^ ^]^ RNA library construction and sequencing were performed on the Illumina HiseqTM 2500/4000 by Gene Denovo Biotechnology Co., Ltd. (Guangzhou, China). The differential expression between given pairwise comparison groups was evaluate by DESeq2 software.^[^ ^35^ ^]^ Bioinformatic analysis such as GO enrichment analysis, pathway enrichment analysis, and GESA were performed using Omicsmart, a real‐time interactive online platform for data analysis.

### Ovarian Peritoneal Disseminated Tumor Model Establishment

The ovarian peritoneal disseminated tumor model using SKOV3 cells was established as previously.^[^ ^36^ ^]^ Briefly, SKOV3 cells (2 × 10^6^) were intraperitoneally injected as a cell suspension into NOD/SCID mice. Five days post inoculation, animals were imaged using the IVIS spectrum system to monitor the tumor progression.

### In Vivo Imaging Study

To study the biodistribution of CaALN nanoparticles in mice, tumor‐bearing mice (*n* = 3) were intraperitoneally injected with CaALN/DiR and free DiR at an equivalent DiR dose of 0.5 mg kg^−1^. The IVIS imaging system was used to acquire real‐time bioluminescence and fluorescence images at 0, 1, 4, 24, and 72 h. Finally, the mice were sacrificed, and major organs and tumor tissue were collected for imaging.

### In Vivo Therapeutic Effect of CaALN

SKOV3 tumor‐bearing mice were randomly divided into three groups (*n* = 8), as follows: PBS, low dose CaALN (10 mg kg^−1^), and high dose CaALN (25 mg kg^−1^). Mice were intraperitoneally injected with drugs for seven weeks. All the mice were imaged using the IVIS Spectrum to monitor the extent of tumor progression. On day 31, three mice of each group were sacrificed. The weight of ascites and tumor weight of each mouse were measured, and the main organs and tumor tissues were extracted for H&E and TUNEL staining. Other mice were treated for seven cycles and fed until death. Animal procedures were reviewed and authorized by the Animal Ethics Committee of Sun Yat‐sen University (SYSU‐IACUC‐2021‐B0815).

### Statistical Analysis

Statistical analysis was performed using SPSS software. Data were presented as the mean ± SD (*n* = 3). The differences between two independent groups were analyzed using *T*‐test. Statistical significance was indicated by *P*‐values as follows. ***P < 0.001, **P <0 .01, *P < 0.05.

## Conflict of Interest

The authors declare no conflict of interest.

## Author Contributions

The manuscript was written through contributions of all authors. All authors have given approval to the final version of the manuscript. J.Z., X.H., and P.L. contributed equally to this work. J.Z., M.Y., and X.H. performed investigation, formal analysis, data curation, software, and writing original draft; P.L. carried out formal analysis, data curation, resources, and validation; B.L., S.L., and M.W. performed investigation, writing – review and editing; M.Q. and Q.W. provided resources and software; Y.C. and Y.P. performed conceptualization, methodology, funding acquisition, project administration, and supervised the study.

## Supporting information

Supporting Information

## Data Availability

The data that support the findings of this study are available from the corresponding author upon reasonable request.
