# Supplementary material for: Engineering Alendronate‐Composed Iron Nanochelator for Efficient Peritoneal Carcinomatosis Treatment
Source: Adv Sci (Weinh). 2022 Sep 4;9(30):2203031. doi: 10.1002/advs.202203031 (PMC9596851; doi:10.1002/advs.202203031)
Supplement: Supplementary file 1 — Supporting Information [file ADVS-9-2203031-s001.pdf]

ADVANCED SCIENCE
Open Access

Supporting Information

for *Adv. Sci.*, DOI 10.1002/advs.202203031

Engineering Alendronate-Composed Iron Nanochelator for Efficient Peritoneal Carcinomatosis Treatment

Jing Zhao, Xiuyu Huang, Peng Liu, Miaojuan Qiu, Binbin Li, Yingfei Wen, Yongshu Li, Qiang Wang, Meiying Wu, Yu Chen* and Yihang Pan**

Supporting Information

Engineering alendronate-composed iron nanochelator for efficient peritoneal carcinomatosis treatment

Jing Zhao†, Xiuyu Huang†, Peng Liu†, Miaojuan Qiu, Binbin Li, Yingfei Wen, Yongshu Li, Qiang Wang, Meiying Wu, Yu Chen*, Yihang Pan**

Prof. J. Zhao, X.Y. Huang, P. Liu, M. J. Qiu, B. B. Li, Q. Wang, Prof. Y. H. Pan
Scientific Research Center, The Seventh Affiliated Hospital of Sun Yat-sen University, Sun Yat-sen University, Shenzhen 518107, P. R. China
E-mail: panyih@mail.sysu.edu.cn

Prof. Y. Chen
Materdicine Lab, School of Life Sciences, Shanghai University, Shanghai, 200444, P. R. China
E-mail: chenyuedu@shu.edu.cn

Prof. M. Y. Wu
School of Pharmaceutical Sciences (Shenzhen), Shenzhen Campus of Sun Yat-sen University, Shenzhen, 518107, Guangdong, P. R. China.
E-mail: wumy53@mail.sysu.edu.cn

Supplementary figures

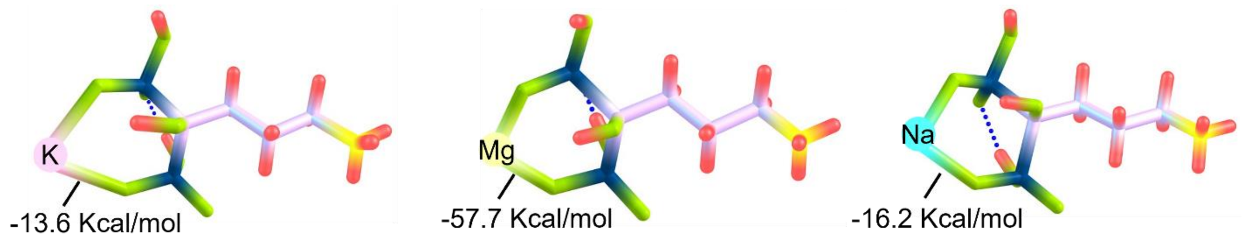

Figure S1. Schematic illustration of coordination between alendronate and K^{+}, Mg^{2+} and Na^{+}.

Element	Weight%
C	53.32
O	23.92
N	4.17
P	9.79
Ca	6.49

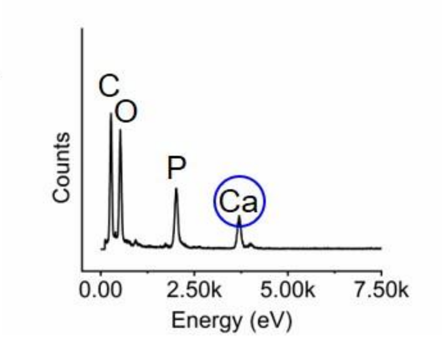

Figure S2. EDX analysis of CaALN.

Element	Weight%
C	68.25
O	15.03
N	4.96
P	6.00
Mn	5.76

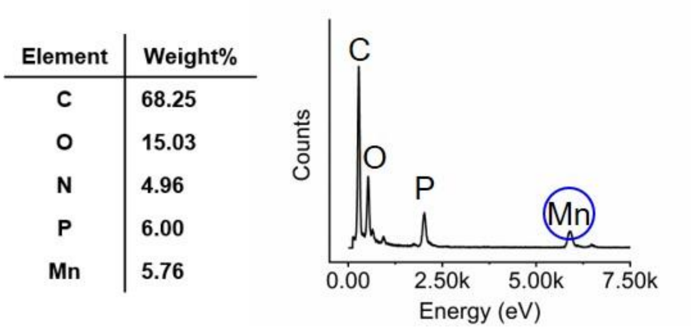

Figure S3. EDX analysis of MnALN.

Element	Weight%
C	76.73
O	12.51
N	2.96
P	4.15
Fe	3.62

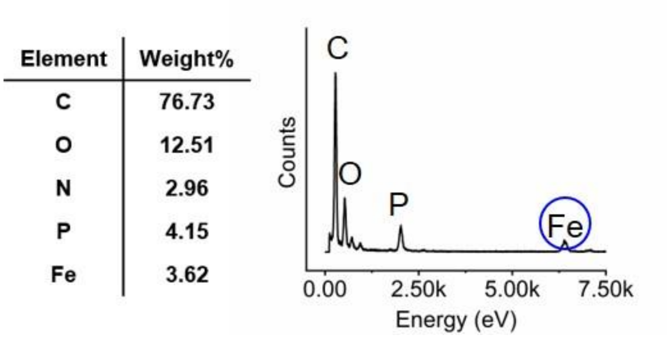

Figure S4. EDX analysis of FeALN.

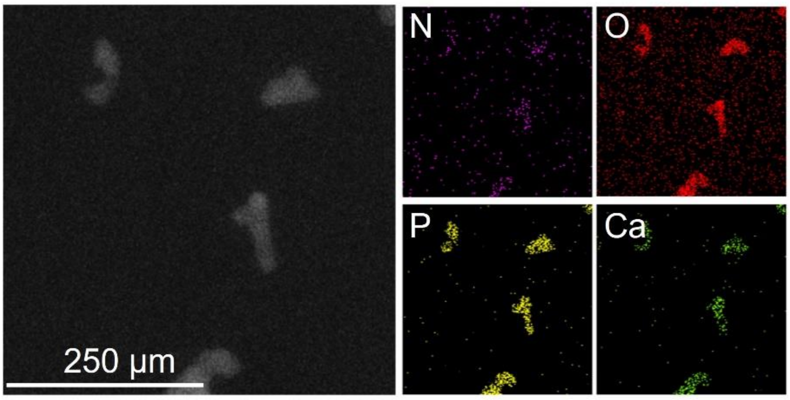

Figure S5. Elemental mapping of CaALN nanoparticles.

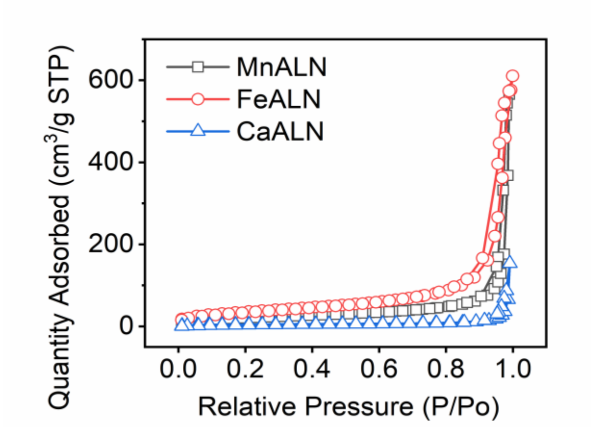

Figure S6. Nitrogen-adsorption-desorption isotherms of ICPs.

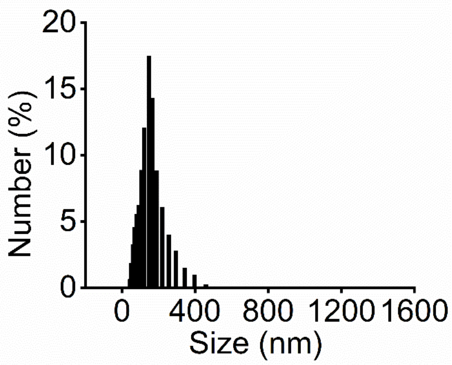

Figure S7. Size distribution of CaALN nanoparticles in water.

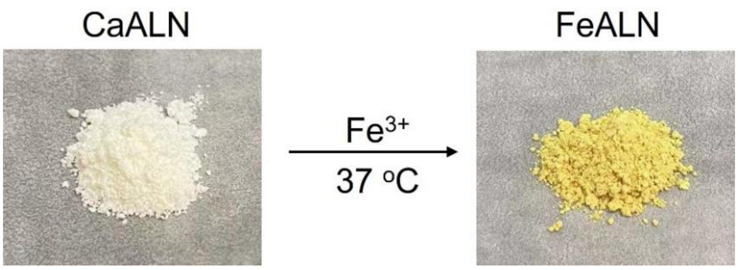

Figure S8. photographs of CaALN powder before and after immersing in $FeCl_3$ solution for 24 hours.

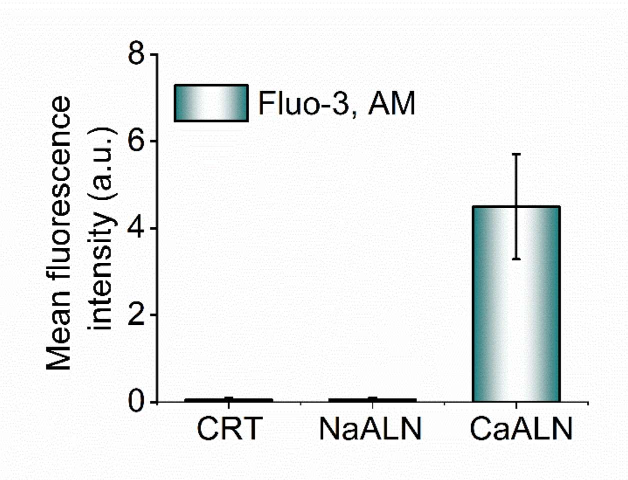

Figure S9. The mean fluorescence intensities of SKOV3 cells treated with NaALN and CaALN at concentration of 100 mg/L for 48 h and stained with Fluo-3 AM.

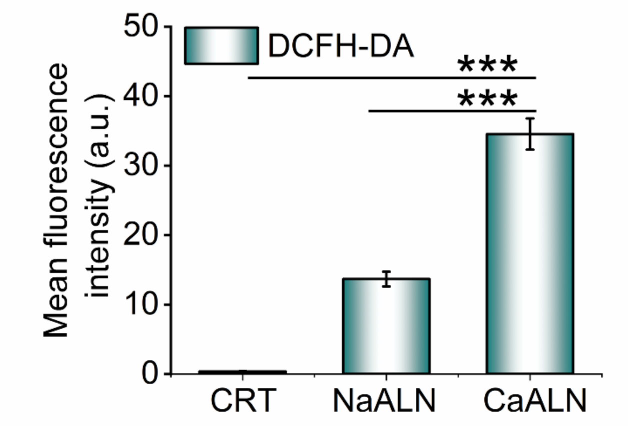

Figure S10. The mean fluorescence intensities of SKOV3 cells treated with NaALN and CaALN at concentration of 100 mg/L for 48 h and stained with DCFH-DA dye (mean ± SD, n = 3, ***$P < 0.001$ versus control group).

.

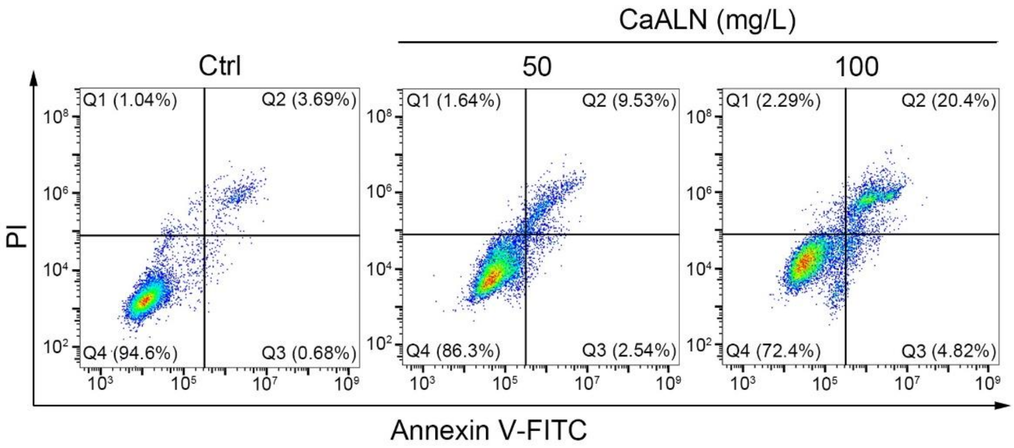

Figure S11 SKOV3 cells were treated with CaALN nanomedicine for 48 h followed by flow cytometry assay with Annexin/ PI staining.

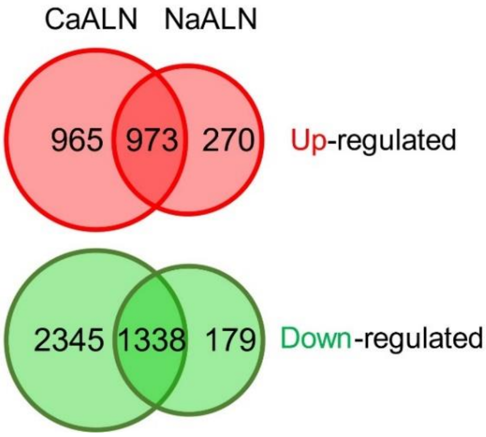

Figure S12. Venn diagrams presenting the number of genes differentially expressed ($p < 0.05$) in cells treated with NaALN and CaALN after FDR multiple testing correction and $\log_2$ (fold change) > 0.75.

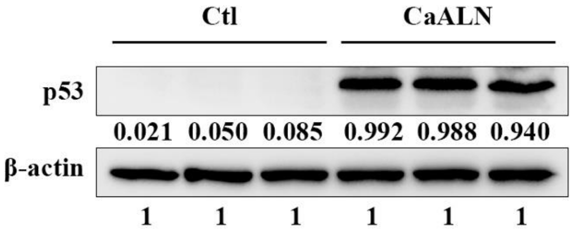

Figure S13. Western blot analysis of SKOV3 cells treatment with CaALN at concentration of 100 mg/L for 48 h.

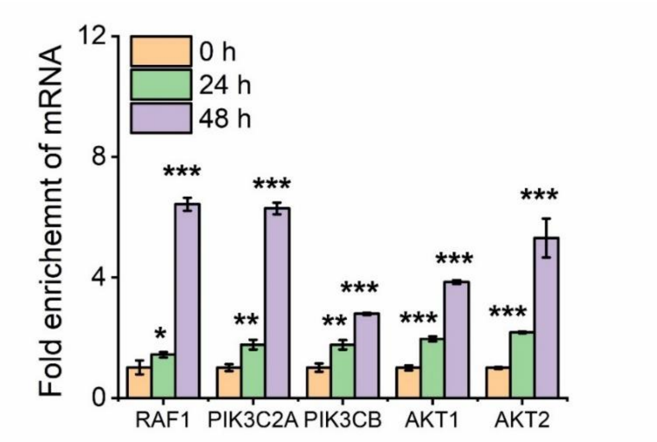

Figure S14. The relative expression of genes on RAS signaling pathway was detected by qRT-PCR (mean ± SD, n = 3, ***$P < 0.001$, **$P < 0.01$ versus 0 h).

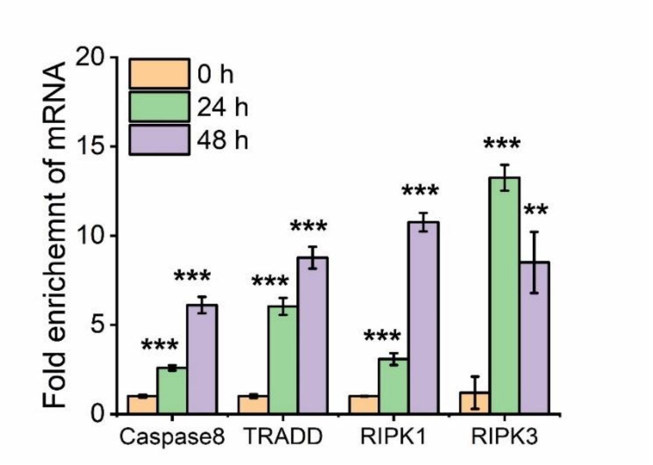

Figure S15. The relative expression of genes on TNF signaling pathway was detected by qRT-PCR (mean ± SD, n = 3, ***$P < 0.001$, **$P < 0.01$ versus 0 h).

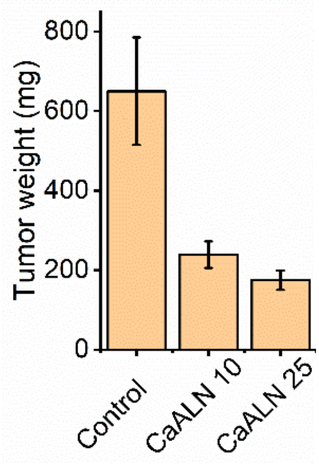

Figure S16. Tumor weight of SKOV3 tumor-bearing mice in PBS and CaLAN groups at day 31.

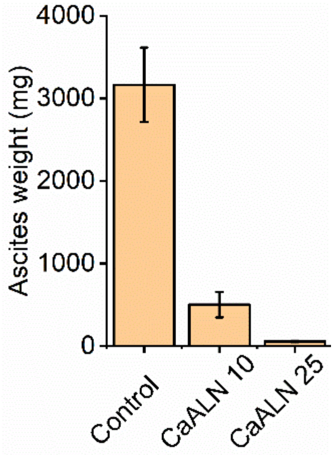

Figure S17. Ascites weight of SKOV3 tumor-bearing mice in PBS and CaLAN groups at day 31.

GENE PRIMERS ENGAGED IN EXPERIMENTS		
ACTB	Forward	5'-TCAAGATCATTGCTCCTCCTGAG-3'
	Reverse	5'-ACATCTGCTGGAAGGTGGACA-3'
casepase-8	Forward	5'-AGAAGAGGGTCATCCTGGGAGA-3'
	Reverse	5'-TCAGGACTTCCTTCAAGGCTGC-3'
TRADD	Forward	5'-ACAAGGTGGTCCTGTCGGATGC-3'
	Reverse	5'-CGGTGGATCTTCAGCATCTGCA-3'
RIPK-1	Forward	5'-TATCCCAGTGCCTGAGACCAAC-3'
	Reverse	5'-GTAGGCTCCAATCTGAATGCCAG-3'
RIPK-3	Forward	5'-GCTACGATGTGGCGGTCAAGAT-3'
	Reverse	5'-TTGGTCCCAGTTCACCTTCTCG-3'
RAF1	Forward	5'-TCAGGAATGAGGTGGCTGTTCTG-3'
	Reverse	5'-CTCGCACCACTGGGTCACAATT-3'
PIK3C2A	Forward	5'-CTTACTCATTGCTTCACCAGTGG-3'
	Reverse	5'-GCCTCAATCCAGGTCACAGCTA-3'
PIK3CB	Forward	5'-GGTAATCGGAGGATAGGGCAGT-3'
	Reverse	5'-CGGCAGTATGCTTCAAGGATGAC-3'
AKT1	Forward	5'-TGGACTACCTGCACTCGGAGAA-3'
	Reverse	5'-GTGCCGCAAAAGGTCTTCATGG-3'
AKT2	Forward	5'-CATCCTCATGGAAGAGATCCGC-3'
	Reverse	5'-GAGGAAGAACCTGTGCTCCATG-3'

Figure S18. The sequences of primers were designed and used for qRT-PCR.
